# Depletion of cardiac catecholamine stores impairs cardiac norepinephrine re-uptake by downregulation of the norepinephrine transporter

**DOI:** 10.1371/journal.pone.0172070

**Published:** 2017-03-10

**Authors:** Michael M. Kreusser, Lorenz H. Lehmann, Markus Haass, Sebastian J. Buss, Hugo A. Katus, Dirk Lossnitzer

**Affiliations:** 1 Department of Cardiology, University of Heidelberg, Heidelberg, Germany; 2 DZHK (German Center for Cardiovascular Research), partner site Heidelberg/Mannheim, Germany; 3 Department of Cardiology, Theresienkrankenhaus, Mannheim, Germany; 4 Department of Cardiology, University Hospital Mannheim, Mannheim, Germany; Universita degli Studi di Napoli Federico II, ITALY

## Abstract

In heart failure (HF), a disturbed cardiac norepinephrine (NE) homeostasis is characterized by depleted cardiac NE stores, impairment of the cardiac NE re-uptake by the neuronal norepinephrine transporter (NET) and enhanced cardiac NE net release. Reduced cardiac NE content appears to be caused by enhanced cardiac NE net release from sympathetic neurons in HF, triggered by neurohumoral activation. However, it remains unclear whether reduced NE itself has an impact on cardiac NE re-uptake, independent of neurohumoral activation. Here, we evaluated whether depletion of cardiac NE stores alone can regulate cardiac NE re-uptake. Treatment of Wistar rats with reserpine (5 mg/kg/d) for one (1d) or five days (5d) resulted in markedly reduced cardiac NE content, comparable to NE stores in experimental HF due to pressure overload. In order to assess cardiac NE re-uptake, the specific cardiac [^3^H]-NE uptake via the NET in a Langendorff preparation was measured. Reserpine treatment led to decreased NE re-uptake at 1d and 5d compared to saline treatment. Expression of tyrosine hydroxylase (TH), the rate-limiting enzyme of the NE synthesis, was elevated in left stellate ganglia after reserpine. Mechanistically, measurement of NET mRNA expression in left stellate ganglia and myocardial NET density revealed a post-transcriptional downregulation of the NET by reserpine. In summary, present data demonstrate that depletion of cardiac NE stores alone is sufficient to impair cardiac NE re-uptake via downregulation of the NET, independent of systemic neurohumoral activation. Knowledge about the regulation of the cardiac NE homeostasis may offer novel therapeutic strategies in HF.

## Introduction

Activation of the sympathetic nervous system is a major characteristic in chronic heart failure (HF) and an elevation of norepinephrine (NE) plasma levels is associated with a poor prognosis in HF patients [1–4]. Under physiological conditions, net cardiac release of NE contributes only minutely to circulating NE [5]. However, the myocardial net secretion of NE in HF increases disproportionately to the degree of systemic sympathetic activation. As a consequence, up to 10% of circulating NE is derived from the failing heart, indicating an excessive local over-activation in the failing myocardium [6]. Even in patients with mild to moderate HF, cardiac spillover of NE is enhanced [7]. In parallel to this sympathetic over-activation, progressive HF is characterized by a paradoxical regression of the cardiac sympathetic nervous system, displayed by the reduction of sympathetic nerve endings and reduced myocardial NE stores. This entails further increased NE in the synaptic cleft leading to enhanced postsynaptic adrenoceptor stimulation, decreased myocardial adrenoceptor density, and subsequent myocyte apoptosis [8–10]. This is well accepted as an important mechanism how neurohumoral activation leads to cardiac remodeling, cardiac dysfunction and arrhythmias in HF.

Cardiac NE homeostasis and myocardial NE net release principally depend on the release and re-uptake of NE, the main transmitter of the sympathetic nervous system [2]. After its release into the synaptic cleft, more than 90% of NE is removed by neuronal re-uptake (uptake_1_) via the sodium-dependent, neuronal norepinephrine transporter (NET), whereas extraneuronal NE re-uptake (uptake_2_) plays only a minor role in the heart [11–13]. Impairment of NET-mediated NE re-uptake in HF markedly increases the "effective" NE concentration at postsynaptic adrenoceptors in the synaptic cleft [8, 14, 15]. We have previously shown that an impairment of NE re-uptake in experimental HF is mediated by posttranscriptional down-regulation of the neuronal NET within the failing heart [11]. This impairment of cardiac NE re-uptake by NET downregulation contributes to an increased cardiac net release of NE in HF, which is associated with the depletion of cardiac NE stores, downregulation of cardiac beta-adrenoceptors and profound alterations of post-receptor signal coupling. This in turn results in adrenoceptor desensitization [8, 16]. Clinical studies have shown that an impaired cardiac NE re-uptake is also associated with a poor prognosis in HF patients, reflected by the worsening of HF and an increasing incidence of sudden death [17, 18]. The distinct mechanisms regulating disturbed NE homeostasis in HF are incompletely understood. In the present study, we followed the hypothesis that the depletion of cardiac NE stores itself may have an impact on presynaptic NE re-uptake and cardiac NE homeostasis. To examine this, we used an established experimental model of HF, transverse aortic constriction (TAC) in rats, in which disturbed NE homeostasis is well described, as well as a model of cardiac NE depletion by reserpine administration. Interestingly, both models displayed comparable alterations regarding NE stores and NE homeostasis. Thus, our data demonstrates that the depletion of NE stores alone leads to a disturbed NE homeostasis, which displays many features seen in HF.

## Materials and methods

### Experimental animals

The present work adheres to the guiding principles of the Declaration of Helsinki regarding ethical conduct of animal research, held by the World Medical Association [19]. In addition, the investigation conforms to the guide for care and use of laboratory animals published by the European legislation (*European Union directive for the protection of animals used for specific purposes 609/1986*, *revised in 2010/63/EU*) and was approved by the responsible regulatory state authorities (Regierungspräsidium Karlsruhe, Germany). The experiments were performed in a total number of 85 male Wistar rats, 8–12 weeks old (Charles River Wiga, Sulzfeld, Germany). The animals were given humane care and were housed in Plexiglas chambers in groups of two. Ad libitum access to standard rodent pellet-diet and water was provided at all times. The laboratory conditions were optimally maintained, in terms of temperature, humidity and light-to-dark cycles.

### Transverse aortic constriction

Pressure overload was induced by transverse aortic constriction (TAC) by partially occluding the ascending aorta with a tantalum clip as described previously [11, 20]. A group of sham-operated rats (surgery without insertion of a clip) served as controls. Rats were anesthetized using intraperitoneal injections of midazolam (5 mg/kg), and fentanyl (0.05 mg/kg). At the end of surgery, anesthesia was reversed by subcutaneous injection of flumazenil (0.5 mg/kg). The rats were kept on a heating plate until recovering from anesthesia and received subcutaneously buprenorphin (60 μg/kg) for further analgesia one hour after surgery. Following surgery, rats were housed individually for 6 days. After six days, the animals were reunited with their cage-mates. Rats were visited and weighed daily during the first two weeks, and every two days thereafter. For long-term analgesia metamizole (1.33 mg/ml) was added to the drinking water. After four weeks, animals were euthanized and measurements were performed.

### Reserpine administration

Reserpine was administrated by daily intraperitoneal injection of 5 mg/kg/d, as previously described [21]. This treatment has been shown to result in effective exhaustion of catecholamine stores in several organs, including sympathetic nerve endings in the heart [22]. Animals were euthanized and measurements were performed after one or five days (1d and 5d) of reserpine treatment.

### Echocardiography

Transthoracic echocardiography was performed four weeks after TAC or sham surgery as previously described in detail [23]. The investigator who conducted the echocardiography was blinded to the treatment status.

### Tissue and organ harvesting

At the indicated time points after TAC/sham surgery or reserpine treatment, rats were anesthetized with thiopental (100 mg/kg; intraperitoneal injection; Byk Gulden, Konstanz, Germany) and euthanized by cervical dislocation. Left stellate ganglia were quickly removed, immediately frozen in liquid nitrogen, and maintained at -80°C until used for RNA extraction. The hearts were excised, weighed and immediately used for isolated heart perfusion, or frozen in liquid nitrogen and maintained at -80°C until NE concentration or NET density was determined. In addition, lungs were excised for measuring lung wet weight.

### Humane endpoints during the study

We used moribund conditions as humane endpoints during the TAC and reserpine studies. Signs of moribundity included a) lack of responsiveness to manual stimulation, b) immobility, and/or c) an inability to eat or drink. In such conditions, animals were euthanized by carbon dioxide asphyxiation.

### Isolated heart perfusion

For isolated perfusion, hearts were prepared according to a modified Langendorff technique, as previously described in detail [11, 24]. The rats were anesthetized with thiopental (100 mg/kg; intraperitoneal injection). The hearts were rapidly cut out, rinsed in ice-cold buffer and the aorta was cannulated for perfusion according to Langendorff. Within one experiment, twelve spontaneously beating hearts were perfused simultaneously at a constant coronary flow and a constant temperature of 37.5°C and all perfusions were performed in the morning hours (7–10 A.M.). The perfusion medium was a modified Krebs-Henseleit solution (composition in mmol/l: NaCl 125, NaHCO_3_ 16.9, Na_2_HPO_4_ 0.2, KCl 4.0, CaCl_2_ 1.85, MgCl_2_ 1.0, glucose 11, EDTA 0.027). The buffer was gassed with 95% O_2_ and 5% CO_2_ and the pH was adjusted to 7.4. Cardiac [^3^H]-NE uptake was determined as described previously [11, 24]. Briefly, a bolus of [^3^H]-NE (1 ml, 3 μCi, 100 pmol NE; Amersham-Buchler, Braunschweig, Germany) was injected into the perfusion system and proportionally distributed to the hearts and blank channels. Radioactivity was measured in the effluent. To avoid artificial findings due to different experimental conditions, we only compared animal groups that were measured together in one single NE re-uptake experiment with one batch of [^3^H]-NE. Experiments were performed in the absence and presence of the NET inhibitor desipramine (DMI, 1 μmol) in order to distinguish between neuronal and extraneuronal NE uptake. The amount of [^3^H]-NE, which was extracted by the hearts (uptake), is expressed as the percentage of radioactivity measured in the blank channels.

### RT-PCR

Total cellular RNA was isolated from frozen left stellate ganglia by acid guanidium thiocyanate/phenol/chloroform extraction [25]. Reverse transcription was performed from left stellate ganglia total RNA using 1st Strand cDNA Synthesis Kit for RT-PCR (Roche Diagnostics, Mannheim, Germany) according to the manufacturer’s instructions. We performed real-time PCR with LightCycler DNA Master Hybridization Probes (Roche Diagnostics). Specific primers for NET, tyrosine hydroxylase (TH) and rat 18S as well as defined concentration of NET, TH and 18S RNA which served as the standards were used, as previously described [20]. Hybridization probes (labeled with fluorescein and LC-red 640) were selected based on the nucleotide sequences of NET, TH and 18S.

### Measurement of NE concentration

Left (LV) and right (RV) ventricular NE content was determined by high-performance liquid chromatography and electrochemical detection as described previously [11, 26].

### Determination of myocardial NET density

Plasma membranes of LV were prepared for [^3^H]-mazindol binding assay as described previously [11, 27]. Radioligand binding assays were performed in a total volume of 250 μl containing 50 μg plasma membranes and increasing concentrations of [^3^H]-mazindol (specific activity, 52.5 Ci/mmol; NEN Life Science Products, Dreieich, Germany) as a specific ligand of the NET. Nonspecific binding was determined by measuring residual binding in the presence of desipramine (100 μmol/l). The incubation was carried out at 30°C and was terminated by rapid vacuum filtration through a MultiScreenHTS-FB filter plate (Millipore Corp., Schwalbach, Germany). All experiments were performed in triplicate. To calculate the binding capacity, the remaining filter radioactivity was determined.

### Statistics

The results are expressed as mean ± SEM. Statistical analysis was performed with the Graph-Pad Prism Software Package Version 5.0 (GraphPad Software Inc., La Jolla, CA). If only two groups were compared in one experiment, statistical comparisons were performed with the unpaired Student’s t-test. Differences between groups, when more than two groups were in the experiment, were tested by one-way ANOVA. If a significant difference was detected by ANOVA, post hoc comparisons by Bonferroni test for multiple comparisons was used to identify the statistically significant differences. In all tests, a probability value of p<0.05 was considered statistically significant.

## Results

### Depleted NE stores and reduced NE re-uptake in experimental HF

**Fig 1:**
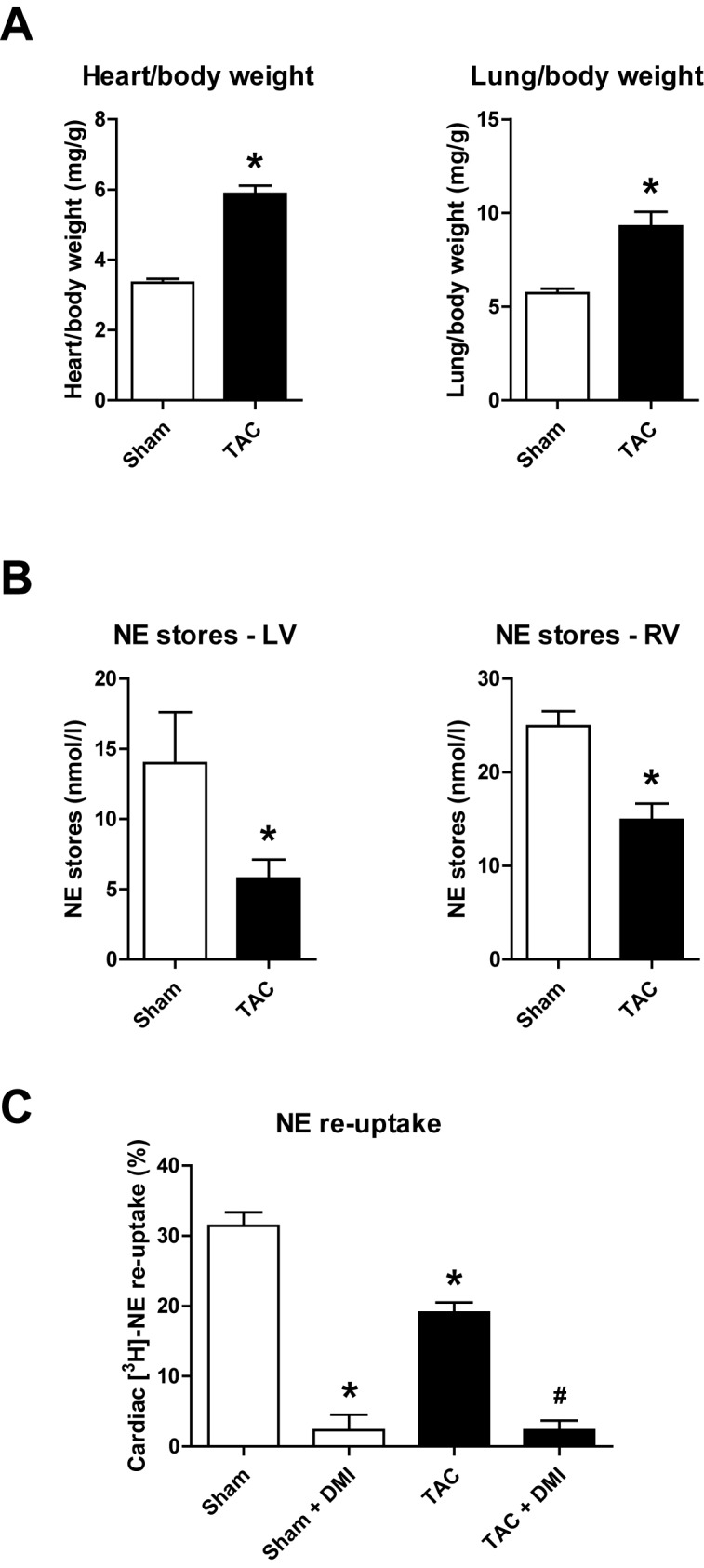
Cardiac norepinephrine (NE) homeostasis after transverse aortic constriction (TAC). **(A)** Heart weight/body weight ratio as a measure for cardiac hypertrophy and increased lung wet weight/body weight ratio as a sign for pulmonary congestion in rats four weeks after TAC (n = 17) or sham (n = 10) operation. **(B)** Left (LV) and right (RV) ventricular NE content after TAC (n = 7) and sham (n = 6) surgery. **(C)** Cardiac [^3^H]-NE uptake in the absence or presence of desipramine (DMI 1 μmol/l) after TAC surgery compared to sham operation (n = 6 per group). *p<0.05 vs. sham; ^#^p<0.05 vs. TAC.

First, we confirmed previous data from our group and others that experimental HF leads to altered cardiac NE homeostasis. We have shown previously that rats challenged with pressure overload due to TAC surgery develop biventricular hypertrophy and overt heart failure with elevated left ventricular end-diastolic pressures and increased plasma proANP level [11, 20]. We also observed in the present study increases in heart weight/body weight and lung wet weight/body weight ratios consistent with our previous studies, indicating cardiac hypertrophy and pulmonary congestion (Fig 1A). Functional data assessed by echocardiography shows elevated systolic and diastolic diameters, left ventricular hypertrophy and cardiac dysfunction after TAC surgery (see Table 1). Moreover, TAC led to markedly decreased NE content (NE stores) in LV and RV tissue (Fig 1B). Consistent with our previous findings, we found a significant reduction of [^3^H]-NE uptake into isolated perfused hearts from TAC animals, indicating a reduced NE re-uptake into sympathetic nerve endings (Fig 1C). There was no difference between TAC and sham rats in the residual uptake of [^3^H]-NE after specific blockade of the NET with DMI, demonstrating that the diminished cardiac elimination of [^3^H]-NE in TAC rats was entirely due to a reduced uptake via the NET, and not, for example, due to the extraneuronal NE transporter (uptake_2_ carrier) (Fig 1C). In summary, our pressure overload HF model displayed altered cardiac NE homeostasis indicated by decreased cardiac NE stores and NE re-uptake via the NET.

**Table 1:** Echocardiography of sham-operated and TAC-operated rats.

	Sham (n = 5)	TAC (n = 5)
Heart rate (bpm)	321.2 ± 20.3	331.8 ± 15.9
LVEDD (mm)	7.0 ± 0.1	8.4 ± 0.5*
LVESD (mm)	4.5 ± 0.6	5.9 ± 1.2
FS (%)	39.4 ± 2.0	28.9 ± 1.1*
LVEF (%)	63.3 ± 3.5	54.6 ± 2.1*
LVPWT (mm)	1.8 ± 0.1	2.7 ± 0.2*
IVST (mm)	1.8 ± 0.1	2.9 ± 0.2*

TAC, transverse aortic constriction; LVEDD, left ventricular end- diastolic dimension; LVESD, left ventricular end-systolic dimension; FS, fractional shortening; LVEF, left ventricular ejection fraction; LVPWT, left ventricular posterior wall thickness; IVST, interventricular septal thickness. Data are given as mean ± SEM.

*p<0.05.

### Effects of one day of reserpine treatment on cardiac NE homeostasis

**Fig 2:**
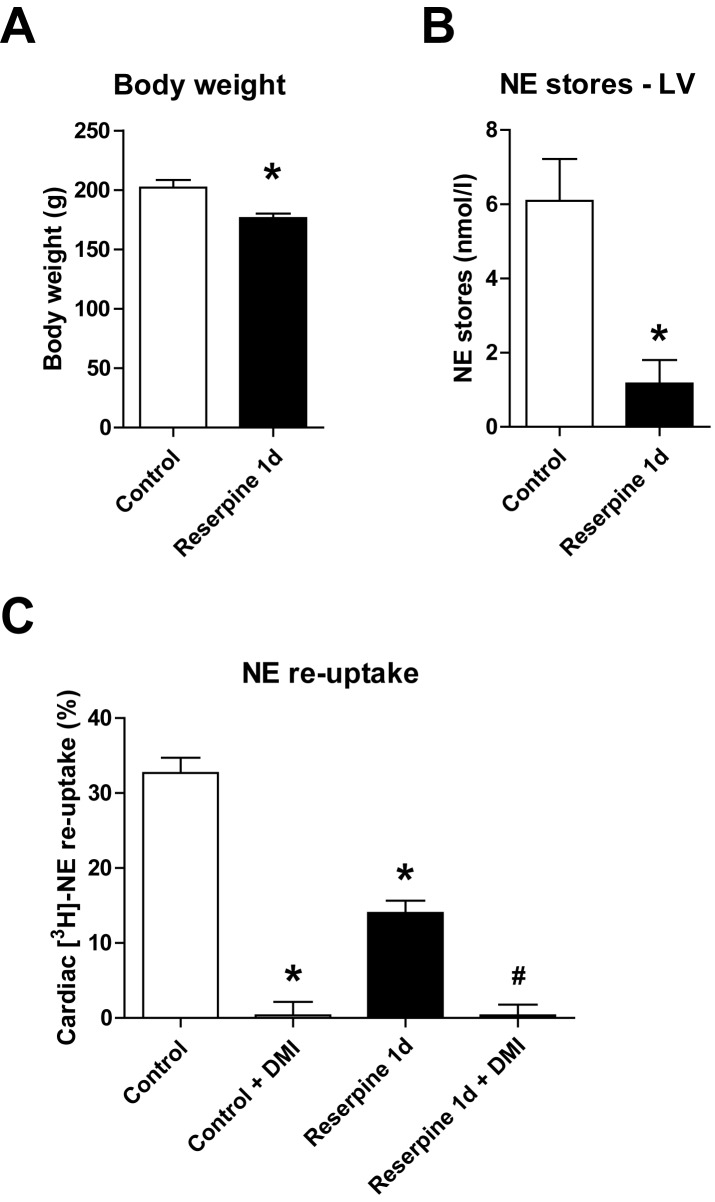
Cardiac norepinephrine (NE) stores and NE re-uptake at day (1d) of reserpine treatment. **(A)** Body weight and **(B)** left ventricular (LV) NE content after reserpine (5 mg/kg) or saline (control) treatment (n = 6 per group). **(C)** Cardiac [^3^H]-NE uptake in the absence or presence of desipramine (DMI 1 μmol/l) after one day of reserpine treatment compared to saline-treated controls (n = 6 per group). *p<0.05 vs. control, ^#^p<0.05 vs. reserpine 1d.

We hypothesized that the depletion of cardiac NE alone was sufficient in regulating NE re-uptake, independent of neurohumoral activation in HF. Treatment with reserpine (1d) led to a slight reduction in total body weight, reflecting the systemic effects of reserpine (Fig 2A), and to reduced cardiac NE stores of less than 20% (Fig 2B). Interestingly and paradoxically, this reduction in cardiac NE stores was accompanied by reduced cardiac uptake of [^3^H]-NE (~40%) in the Langendorff perfusion, indicating a reduced sympathetic NE re-uptake in reserpine-treated animals (Fig 2C). Moreover, there was no difference between reserpine-treated and control rats in the residual uptake of [^3^H]-NE after specific blockade of the NET with DMI, suggesting that the diminished cardiac elimination of [^3^H]-NE was completely mediated by the NET (Fig 2C).

### Cardiac NE homeostasis after five days of reserpine

**Fig 3:**
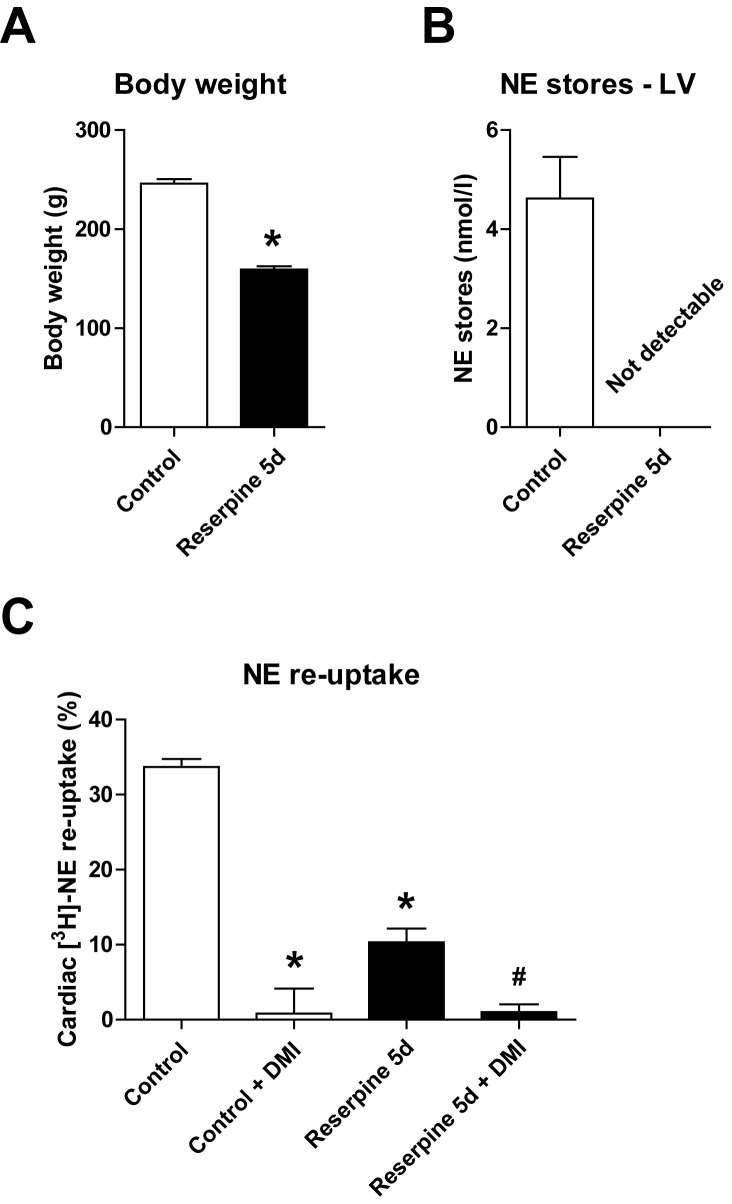
Cardiac norepinephrine (NE) stores and NE re-uptake after five days (5d) of reserpine treatment. **(A)** Body weight and **(B)** left ventricular (LV) NE content after five days of reserpine (5 mg/kg/d) or saline (control) treatment (n = 6 per group). **(C)** Cardiac [^3^H]-NE uptake in the absence or presence of desipramine (DMI 1 μmol/l) after five days of reserpine treatment compared to saline-treated controls (n = 6 per group). *p<0.05 vs. control, ^#^p<0.05 vs. reserpine 5d.

Prolonged treatment with reserpine over five days (5d) led to an even further reduction in total body weight (Fig 3A), and cardiac NE was no longer detectable with our assay (Fig 3B), reflecting the dramatic systemic and cardiac effects of reserpine treatment. Consequently, we found a more pronounced reduction (~70%) in cardiac uptake of [^3^H]-NE in animals treated with reserpine for five days (Fig 3C). Again, specific blockade of the NET with DMI demonstrated that the diminished cardiac elimination of [^3^H]-NE was entirely mediated by the NET (Fig 3C).

### Post-transcriptional regulation of the NET

**Fig 4:**
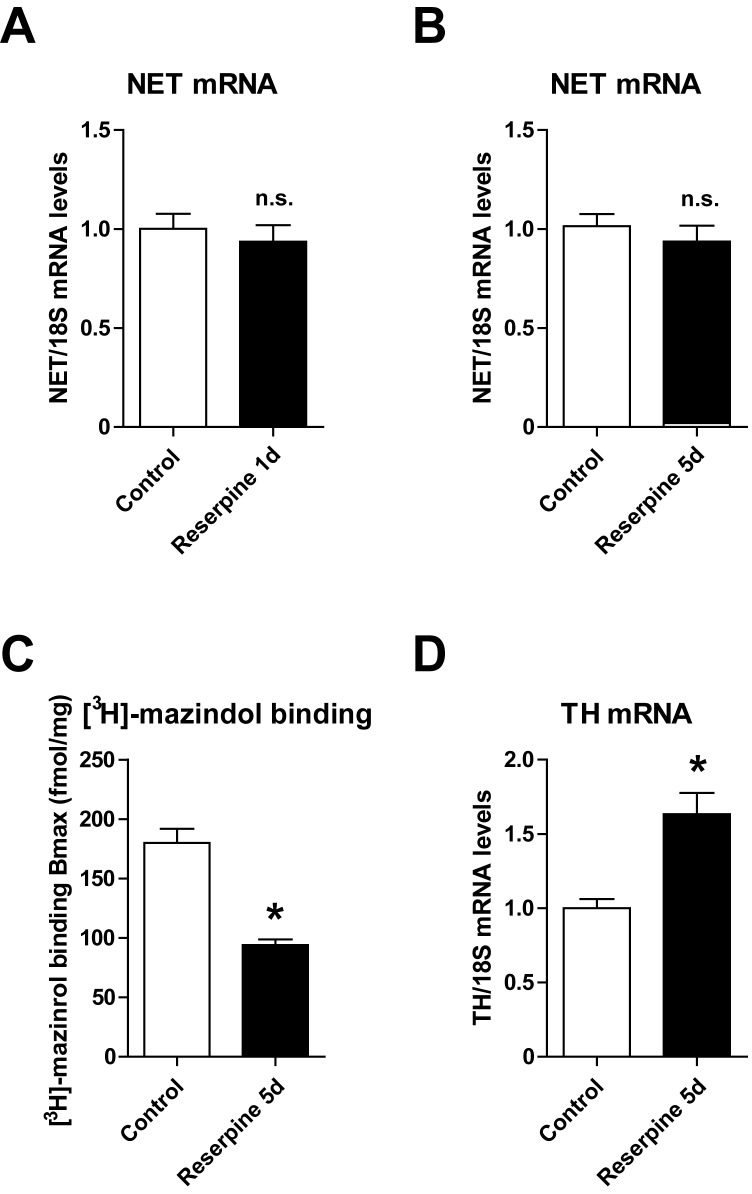
Downregulation of the norepinephrine (NE) transporter (NET) after NE depletion. Gene expression of the NET after one day (1d) **(A)** and five days (5d) **(B)** of treatment with reserpine (5 mg/kg/d) or saline (control), normalized to 18S mRNA expression, respectively (n≥5 for each group). **(C)** Myocardial density of the NET measured as binding of [^3^H]-mazindol to cardiac membranes (normalized to mg total protein; n = 6 per group) and **(D)** expression of the tyrosine hydroxylase (TH) (normalized to 18S mRNA expression; n≥5 for each group) in controls and after reserpine treatment for five days. *p<0.05 vs. control, n. s. = not significant vs. control.

We previously demonstrated that reduced NET function in experimental HF is mediated by a post-transcriptional mechanism [11, 20, 28]. Intriguingly, we did not find a transcriptional regulation of the NET in the stellate ganglia, neither after one nor after five days of reserpine treatment (Fig 4A and 4B). However, a specific NET binding assay using [^3^H]-mazindol measured a lower presence of the NET on the plasma membrane, indicating a post-transcriptional downregulation of the NET after reserpine, comparable to what is described in HF (Fig 4C) [11]. Thus, one could speculate that this post-transcriptional downregulation of the NET may be mediated in both conditions, in HF and after cardiac NE depletion, by similar mechanisms. In addition, the rate-limiting enzyme of norepinephrine synthesis in sympathetic neurons, the TH, was significantly upregulated after reserpine, comparable to what is seen in HF models (Fig 4D) [20, 29].

## Discussion

Our data demonstrate that the depletion of cardiac NE stores leads to changes in cardiac NE that resemble the findings in HF namely, a decreased cardiac NE re-uptake mediated by post-transcriptional downregulation of the NET and, possibly as compensation, an upregulation of TH, the rate-limiting enzyme of NE synthesis. To our knowledge, this is the first study that directly connects myocardial NE content to changes in NE homeostasis independent of HF and HF-associated activation of the neurohumoral system.

### Model of NE depletion

But is reserpine an appropriate model to study the depletion of cardiac NE stores? Reserpine irreversibly blocks the vesicular monoamine transporter that normally transports norepinephrine and other monoamines in the presynaptic nerve terminal to presynaptic vesicles for subsequent release into the synaptic cleft [30]. It has been used in a multitude of studies as a model for catecholamine depletion [21, 31, 32]. In our study, reserpine treatment led to a significant decrease in body weight even after a single administration. The body weight further declined until the end of the observation time after five days. A decrease of body weight greater than 5% was described as a marker for sufficient NE depletion [21] and is part of the reserpine syndrome including ptosis, miosis, lacrimation, salivation, diarrhea, hypotension and sedation. Consistently, NE stores in the LV were significantly reduced after a single day of reserpine treatment and were no longer detectable after five days of reserpine injections. This is once again in accordance with a NE depletion of 75–90% described by others [21]. Thus, we believe that the reserpine model used in our study can be taken as a reliable model of cardiac NE depletion. However, the systemic effects and/or off-target effects of reserpine must be considered when interpreting the data.

### NE homeostasis and NET function

As a sympathetic response to NE depletion, we detected an increased level of mRNA expression of TH in the stellate ganglia, the enzyme that initiates NE biosynthesis by hydroxylation of tyrosine to dopa [29]. We interpret this as an indicator of an enhanced synthesis of NE in response to the loss of cardiac catecholamines under reserpine. However, elevated TH is also seen as part of the disturbed NE homeostasis complex witnessed in HF [20, 29].

The central finding of our study is that we observe a significant reduction of cardiac NE re-uptake not only in the HF but also in the reserpine model. Reserpine was previously shown to impair NE-re-uptake in the cerebral cortex and adrenal glands [33]. However, our study is the first to demonstrate this effect in the myocardium. To determine the specificity of the NE re-uptake measurements, we used DMI as a selective NET antagonist which reduced the level of NE uptake to an almost undetectable level in both, TAC as well as reserpine-treated animals. This indicates that the NET function was primarily affected by reserpine treatment (and TAC surgery) rather than other mechanisms of NE re-uptake such as the extraneuronal uptake_2_ carrier. This is in accordance with many other studies that have identified NET-dependent mechanisms causal for impaired NE re-uptake in cardiac disease models [8, 11, 34]. What then are the molecular mechanisms that underlie this regulation of the NET? We measured unaltered mRNA expression but observed reduced NET density on myocardial plasma membranes after reserpine by using a [^3^H]-mazindol binding assay. Mazindol specifically binds to the NET and this assay presents a valid measurement of NET density on the surface of cardiac sympathetic neurons when plasma membrane preparations are used [11, 27]. This is a more appropriate method for measuring NET expression than general protein levels, because NET is functionally regulated by plasma membrane translocation [11, 35]. Although we cannot distinguish from our data the post-transcriptional level on which NET is regulated (e.g., protein translation, trafficking), we conclude that downregulation of the NET on the plasma membrane is mediated by a post-transcriptional mechanism. In part, this is contrary to findings published by Mandela and colleagues, who investigated the effects of reserpine on NET function in PC12 cells [36]. The authors likewise found that reserpine impaired NE re-uptake, but this was attributed to a direct inhibition of the NET by reserpine-induced loss of storage vesicles and/or blockade of the vesicular monoamine transporter rather than post-transcriptionally. We did not further study how post-transcriptional regulation of the NET was caused on a molecular level. Possible mechanisms include i) impaired mRNA translation into protein, and ii) impaired NET trafficking or insertion into the plasma membrane. An intriguing recent study demonstrated how regulation of NET on a translational level is mediated by microRNAs 29b and 181a as part of the stress response in sympathetic neuron-like PC12 cells [37]. Trafficking or insertion of NET may be regulated by posttranscriptional modification of the protein. For instance, N-glycosylation and direct phosphorylation of the NET by protein kinases (such as protein kinase C) have been discussed as regulatory mechanisms in experimental HF where neurohumoral systems are activated [38–40]. Other kinases (e.g., G protein-coupled receptor kinase 2, GRK2), which are differentially regulated in sympathetic and/or chromaffin cells during HF, can be proposed as potential candidates that may directly regulate NET in HF [16, 41]. Moreover, Kaludercic et al. demonstrated that the reactive oxygen species produced by the monoamine oxidase A may be critical in the regulation of NET during HF [42]. This study implies that oxidative stress may be a direct (potential posttranscriptional modification) or indirect regulator of NET. However, as the NE depletion model we used attenuates the sympathetic tone and thereby is independent of neurohumoral activation, disturbances of the NE homeostasis in HF may be triggered by various mechanisms, neurohumoral-dependent and -independent, and the latter may be induced by depleted NE stores in HF.

### Neurohumoral regulation of NE homeostasis

The concept that activation of the neurohumoral system leads to disturbed NE homeostasis in HF is well established. Given the sympathetic over-activation in HF [3], it has been shown that its own neurotransmitter NE has detrimental effects on cardiac NE homeostasis, and that this is mediated, at least in part, by cytotoxic effects of NE [42–44]. Other components of the activated neurohumoral system, such as the renin-angiotensin-aldosterone-system, have been demonstrated to regulate NE re-uptake and NE net release [23, 45, 46]. Recently, endothelin was established as an important regulator of sympathetic innervation of the heart [47, 48] and NE re-uptake via the NET in experimental HF [27, 34]. Moreover, neurotrophic factors have been discussed as regulators of cardiac NE re-uptake and NE homeostasis in HF [20, 28, 49]. Considering this data on the diverse neurohormone-dependent mechanisms that determine NE homeostasis in HF, it is striking that in our study, the depletion of NE stores alone led to reduction of cardiac NE re-uptake. This observation is in agreement with another study which demonstrated that the depletion of cardiac NE stores by reserpine leads to cardiac dysfunction [50] and may provide support for the idea that depletion of cardiac NE stores in HF itself contributes to the *circulus vitiosus* of chronic heart disease.

### Clinical implications

Heart failure is a pandemic disease with increasing incidence, economic importance and rising morbidity and mortality [51]. Myocardial NE re-uptake is known to be a strong prognostic marker for overall mortality in HF and likewise, an improved NE re-uptake might decrease NE concentration in the synaptic cleft and thereby improve beta-adrenergic signalling. Up to now, only postsynaptic inhibition of NE signalling has been established in the treatment of HF, such as in the use of beta-receptor antagonists. However, modulation of the cardiac NE homeostasis at the presynaptic side of neurotransmission might be a promising and currently underestimated therapeutic approach to HF and other cardiovascular diseases.

### Conclusions

In summary, we demonstrate that the depletion of cardiac NE stores alone impairs cardiac NE re-uptake via post-transcriptional downregulation of NET, independent of systemic neurohumoral activation. We believe that a deeper knowledge about the regulation of the cardiac NE homeostasis may offer novel therapeutic strategies for HF in the future.

## Abbreviations

DMI: desipramine
HF: heart failure
LV: left ventricular
NE: norepinephrine
NET: neuronal norepinephrine transporter
RV: right ventricular
TAC: transverse aortic constriction
TH: tyrosine hydroxylase
